# The *Borreliella burgdorferi* BosR-associated small non-coding RNA BasA regulates virulence

**DOI:** 10.1128/iai.00026-26

**Published:** 2026-03-10

**Authors:** Brittany L. Shapiro, Prashant Jaiswal, Lisa J. Funkhouser-Jones, Sourav Roy, Taylor J. Van Gundy, Jeremy M. Bono, Meghan C. Lybecker, Brandon L. Garcia, Jenny A. Hyde, Jon T. Skare

**Affiliations:** 1 Department of Microbial Pathogenesis and Immunology, Texas A&M University 14736, Bryan, Texas, USA; 2 Department of Biochemistry and Molecular Biophysics, Kansas State University 5308, Manhattan, Kansas, USA; 3 Bacterial Diseases Branch, Division of Vector-Borne Diseases, Centers for Disease Control and Prevention, National Center for Emerging and Zoonotic Infectious Diseases 1242, Fort Collins, Colorado, USA; 4 Department of Biology, University of Colorado at Colorado Springs, Colorado Springs, Colorado, USA; University of California Davis, Davis, California, USA

**Keywords:** *Borreliella burgdorferi*, Lyme disease, gene regulation, post-transcriptional regulation, sRNA

## Abstract

Post-transcriptional regulation is an adaptive response used by living systems to alter protein levels independently of transcription initiation. In bacteria, one mechanism of post-transcriptional regulation involves the binding of small, non-coding RNAs (sRNAs) to target mRNAs to regulate their translation into protein. This regulation generally includes an RNA-binding protein that facilitates the sRNA::mRNA interaction. In the etiological agent of Lyme disease, *Borreliella burgdorferi*, over 400 temperature-dependent sRNAs have been identified. Here, we characterized the sRNA SR0735, which is upregulated at 37°C (characteristic of mammalian infection). Transcriptomic and proteomic analyses revealed that deleting SR0735 in *B. burgdorferi* resulted in reduced production of virulence-associated, RpoS-dependent proteins due to a reduction of RpoS and its upstream regulator BosR. We thus renamed SR0735 as BasA for BosR-associated sRNA locus A. Consistent with previous studies that identified BosR as an RNA-binding protein, we demonstrate here that BosR can bind BasA *in vitro*. Importantly, the loss of BasA significantly attenuated *B. burgdorferi* infectivity during murine infection, which is consistent with the reduced levels of BosR and RpoS observed. The emerging model suggests that BasA is required for optimal levels of BosR protein through the post-transcriptional regulation of *bosR* mRNA, which, in turn, promotes the production of RpoS and its downstream genes required for mammalian infectivity and pathogenesis.

## INTRODUCTION

*Borreliella burgdorferi*, the etiologic agent of Lyme disease, is the most common arthropod-borne infection in the United States, with an estimated 476,000 cases diagnosed each year (1–4). Lyme disease is a multi-stage inflammatory infection characterized by a flu-like illness and, in most instances, a skin lesion known as erythema migrans (3, 5, 6). If untreated, Lyme disease can result in arthritis, carditis, or neurological symptoms (3). *B. burgdorferi* is maintained in its enzootic cycle by *Ixodes scapularis* ticks that acquire the bacterium from its predominant reservoir, the *Peromyscus* mouse (7–10). Infected *Ixodes* ticks can then transmit *B. burgdorferi* to humans (3, 7, 8). Early diagnosis and antibiotic treatment generally resolve *B. burgdorferi* infection; however, some patients treated for Lyme disease will experience post-treatment Lyme disease syndrome (PTLDS), a condition associated with significant morbidity (3, 11, 12). Given the increased incidence of Lyme borreliosis and the pathology associated with chronic infections, a better understanding of how *B. burgdorferi* establishes and maintains infection is important for identifying new therapeutic targets to mitigate Lyme disease.

It is well established that *B. burgdorferi* dramatically alters gene regulation during the tick blood meal when the spirochetes are transmitted from *Ixodes* ticks to vertebrates (7, 8, 13–17). During this transition, RpoS is produced and activates the expression of genes required for mammalian infection (18–22). The expression of *rpoS* is dependent on the BosR regulator, an atypical Fur family member that is able to autoregulate (23–25). BosR levels are enhanced during conditions that mimic transmission into mammals and are required to produce RpoS (25–27). Consistent with its association with RpoS levels, *B. burgdorferi* lacking *bosR* synthesizes significantly less RpoS-regulated virulence-associated transcripts and their encoded proteins, and, as such, *B. burgdorferi* lacking *bosR* is non-infectious (23, 25, 28). Despite these findings, details are lacking as to how BosR regulates RpoS production.

Although initially characterized as a transcriptional regulator (18, 23–25, 29–32), BosR also binds RNA *in vivo* and *in vitro* (33, 34) and has been shown to have RNA chaperone activity *in vitro* (34). One mechanism of trans-encoded small RNA (sRNA) post-transcriptional regulation occurs via sRNA::mRNA base pairing, which is often facilitated by an RNA chaperone (35–39). In several instances, sRNAs have been implicated in regulating virulence-associated bacterial gene expression in response to environmental cues (38, 40–45). Popitsch et al. identified a set of sRNAs whose expression is temperature-dependent in *B. burgdorferi*, implicating borrelial sRNAs in host adaptation (36, 46, 47).

Herein, one of the sRNAs that is upregulated at 37˚C, designated as SR0735, was selected for further characterization (46). Deletion of SR0735 in *B. burgdorferi* resulted in a reduction of BosR, RpoS, and RpoS-regulated proteins. Additionally, BosR was found to bind SR0735 *in vitro*. Loss of SR0735 resulted in significantly attenuated infectivity in the murine model of Lyme borreliosis that mostly phenocopies a *bosR* mutant (25, 27). Given the association of SR0735 with BosR and its effect on BosR production, we have renamed this sRNA BasA for BosR-associated sRNA locus A. Collectively, this work suggests that BasA, together with BosR, post-transcriptionally regulates the *bosR* transcript and subsequent BosR protein levels. The resulting increased levels of BosR drive the activation of *rpoS* and the expression of RpoS-regulated genes required for borrelial infection and pathogenesis.

## RESULTS

### Transcriptomics reveals a global dysregulation of *rpoS* and RpoS-regulated genes in the absence of BasA

**Fig 1:**
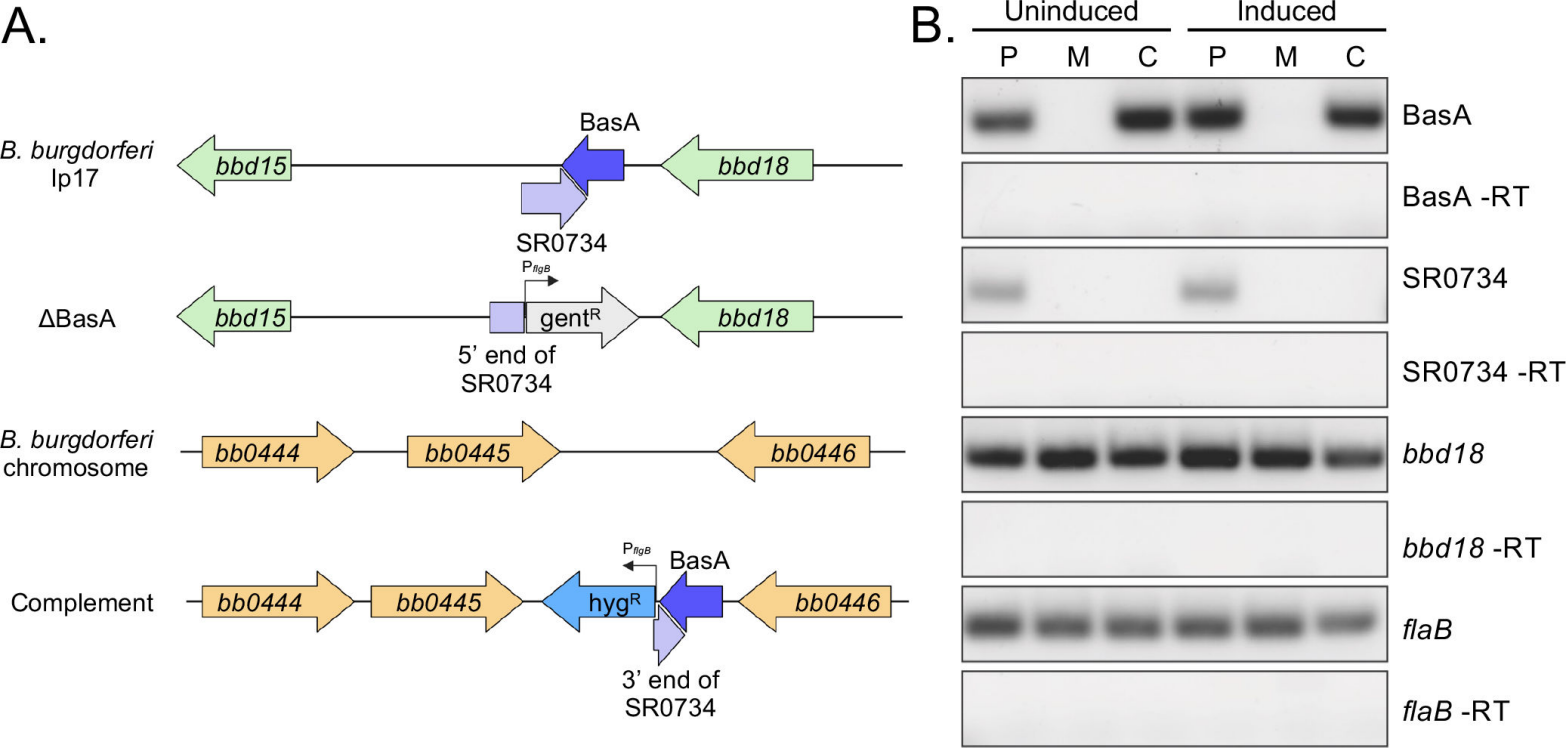
Strategy to genetically inactivate BasA and confirmation of constructs. (**A**) Schematic representation of the BasA (85 bp) deletion and complementation strategy. BasA (dark blue arrow) in its native location on lp17 in the parent strain 5A4 NP1 is shown on the top. A P*_flgB_*-gent^R^ cassette replaced BasA, generating the mutant strain ΔBasA. BasA was restored into the chromosomal site of the mutant with a P*_flgB_*-Hyg^R^ cassette, generating the complement strain. Note that genes, sRNAs, and distances between loci are not drawn to scale. Created in BioRender. Shapiro, B. (2026) https://BioRender.com/2lm8az6. (**B**) Reverse transcriptase PCR (RT-PCR) was used to detect RNA specific for BasA*,* SR0734*, bbd18,* and *flaB* in the strains generated (P = parent, M = ΔBasA mutant, C = complement) grown under both uninduced and induced conditions. “-RT” indicates PCR for BasA, SR0734, or *flaB* without reverse transcriptase as a control for DNA contamination.

Prior work in our group characterized an sRNA known as SR0736, subsequently renamed *ittA*, that exhibited an attenuated phenotype in a mouse infection (47). Here, we characterize an 85 base pair sRNA located near *ittA*, SR0735, which we have renamed as BasA. Previously, BasA was characterized as a temperature-regulated sRNA that was more abundant at higher temperatures (37°C) that mimic a mammalian infection, relative to 23°C (46). Given its upregulation when exposed to higher temperatures, we reasoned that BasA might play an important role in *B. burgdorferi* infectivity within a mammalian host. To investigate the effect of BasA on borrelial pathogenesis, we used homologous recombination to fully delete BasA within the 17 kilobase linear plasmid (lp17) of strain B31 5A4 NP1 that overlaps the 3′ end with SR0734 (Fig. 1A) (48). We then genetically complemented BasA into an established chromosomal site between *bb0445* and *bb0446* in the ΔBasA mutant (Fig. 1A) (49). The complement was designed to restore BasA expression to assess its effects independent of SR0734. All strains generated were screened to ensure that they maintained the plasmid content of the parental strain. Reverse-transcriptase PCR (RT-PCR) confirmed the loss of BasA RNA in the mutant and its restoration in the complement strain under both conventional growth conditions (“uninduced” 32°C, pH 7.6, 1% CO_2_) or conditions that induce genes essential for borrelial mammalian infectivity (“induced” 37°C, pH 6.8, 5% CO_2_) (Fig. 1B). Deletion of BasA did not impact *in vitro* growth under uninduced conditions, but ΔBasA and the complement grew marginally faster than the parent strain under induced conditions (Fig. S1). Regardless, all strains reached a comparable level of growth saturation after 7 days (Fig. S1).

**Fig 2:**
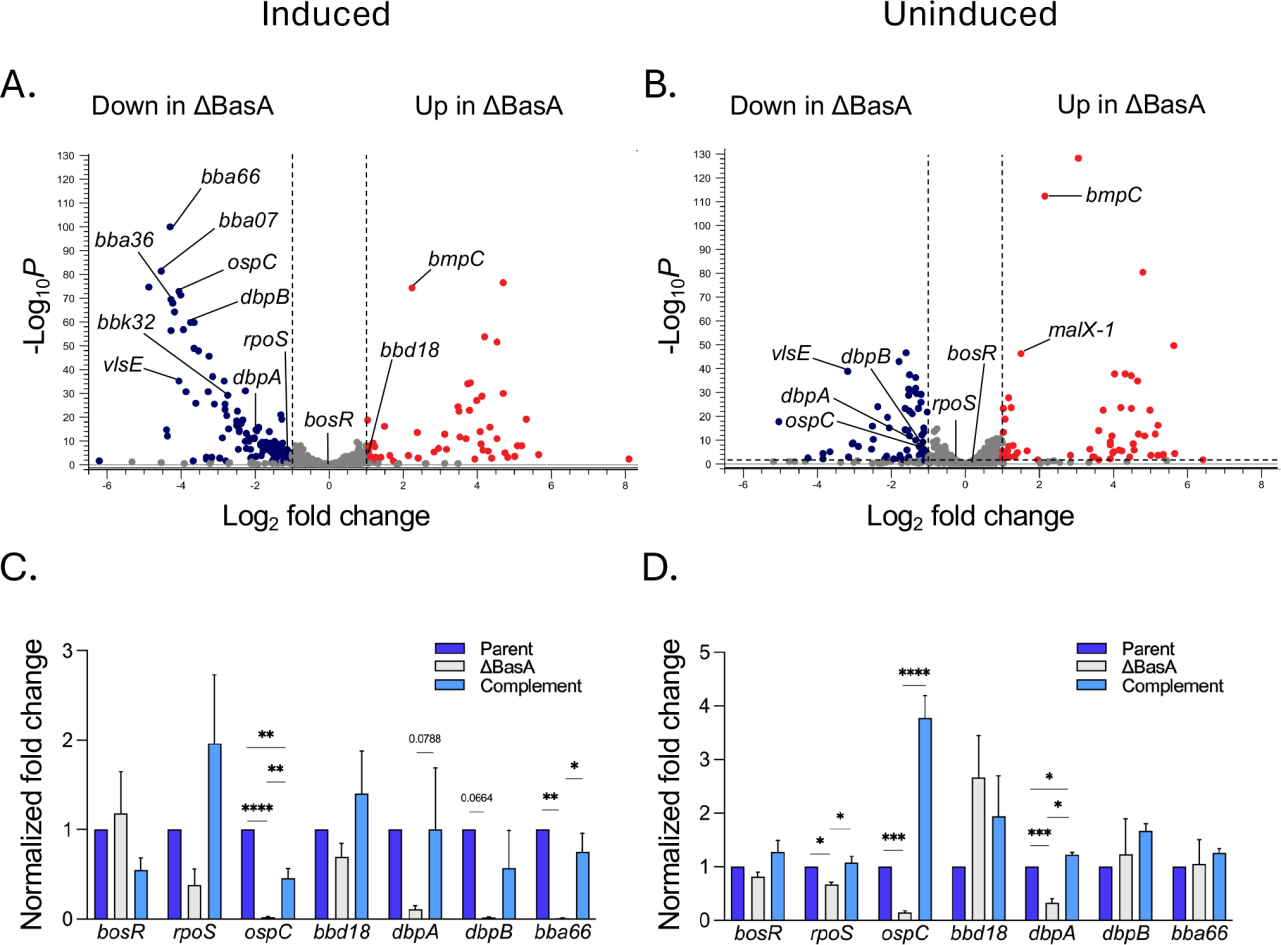
Deletion of BasA alters *B. burgdorferi* transcriptional profiles relative to the parent. Volcano plot depicting differential expression in the ΔBasA mutant relative to the parent grown under induced conditions is shown in (**A**) and for uninduced conditions in (**B**). Log_2_ fold change in transcript abundance is plotted on the x-axis, and the FDR *P*-value is plotted on the y-axis for both comparisons. Dots on the volcano plot represent individual genes. Transcripts that met the criteria for statistical significance (FDR-corrected *P*-value <0.05 with an absolute log_2_ fold change >1) are labeled in blue and red for down and upregulated, respectively. Reverse transcriptase quantitative PCR (RT-qPCR) of the parent (dark blue), mutant (gray), and complement (light blue) strains for transcripts indicated at the bottom of each panel using cells grown under induced conditions in (**C**) and uninduced conditions in (**D**). For both comparisons, all transcripts were normalized to a *flaB* control. The ratio of the target gene relative to *flaB* transcripts is scored as 1.0 for the parent strain. Error bars represent standard error of the mean (SEM). Significance is denoted as *P < 0.05, **P < 0.01, ***P < 0.001, ****P < 0.0001, determined using a one-way ANOVA with a Tukey’s comparison.

Because BasA is upregulated at higher temperatures (46), consistent with products involved in borrelial mammalian infectivity, we hypothesized that BasA could be involved in the regulation of transcripts essential for mammalian infectivity. Thus, we used RNA sequencing to examine transcriptional profiles of the ΔBasA mutant relative to the parent and complement under induced and uninduced conditions. When grown under induced conditions, a total of 108 genes were significantly differentially regulated (absolute log_2_ fold change > 1 and a false discovery rate [FDR]-corrected *P*-value < 0.05) in the ΔBasA mutant compared to the parent strain (Fig. 2A; Table S2). Of these 108 genes, 88 were downregulated and 20 upregulated in the ΔBasA mutant relative to the parent (Fig. 2A; Table S2). Overall, 69% of the downregulated genes in the ΔBasA mutant are regulated by BosR and RpoS, including *ospC*, *dbpA*, *dbpB*, and *bba66* (19, 21, 28, 50–54). Additionally, 35% of the upregulated genes in the ΔBasA mutant are also regulated by BosR and RpoS, including *bmpC* (21, 28). Within this analysis, *rpoS* and *bbd18* transcript levels were downregulated 2.1-fold and upregulated 2.1-fold, respectively. This trend for *rpoS* and *bbd18* is consistent with the known regulatory effects of BosR (23, 25, 28).

Under uninduced growth conditions, when BosR and RpoS production are low (18, 23, 25, 26), the loss of BasA had a more subtle effect on global gene dysregulation, with only 33 genes (Fig. 2B; Table S2) significantly downregulated in the ΔBasA mutant relative to the parent. Nine of these genes, including *ospC*, *vlsE*, and *dbpB*, are also significantly downregulated in the ΔBasA mutant relative to the parent under induced growth conditions, while 24 genes are uniquely downregulated in the uninduced growth condition.

Expression of select genes observed in the RNA-seq analysis (*bosR*, *rpoS*, *ospC*, *bbd18*, *dbpA*, *dbpB*, and *bba66*) was validated by RT-qPCR in the parent, ΔBasA mutant, and complement strains relative to *flaB* under induced (Fig. 2C) and uninduced conditions (Fig. 2D). Interestingly, no differences in *bosR* transcript levels were observed between the parent, mutant, and complement strains in both conditions (Fig. 2C and D), consistent with prior work that found mammalian-like inducing conditions did not alter *bosR* transcripts (26).

Chromosomal complementation of BasA restored 65% of transcripts dysregulated by the loss of BasA under induced conditions and 77% when cells were grown under uninduced conditions (Fig. S2A and B; Table S2). Expression differences observed between parent and complement strains can largely be attributed to genes on lp36 and cp32-6 plasmids (Fig. S2A and B; Table S3), which may have been lost in a subset of the complement and parent populations, respectively.

### Proteomics reveals a global dysregulation of BosR and RpoS-regulated proteins in the absence of BasA

**Fig 3:**
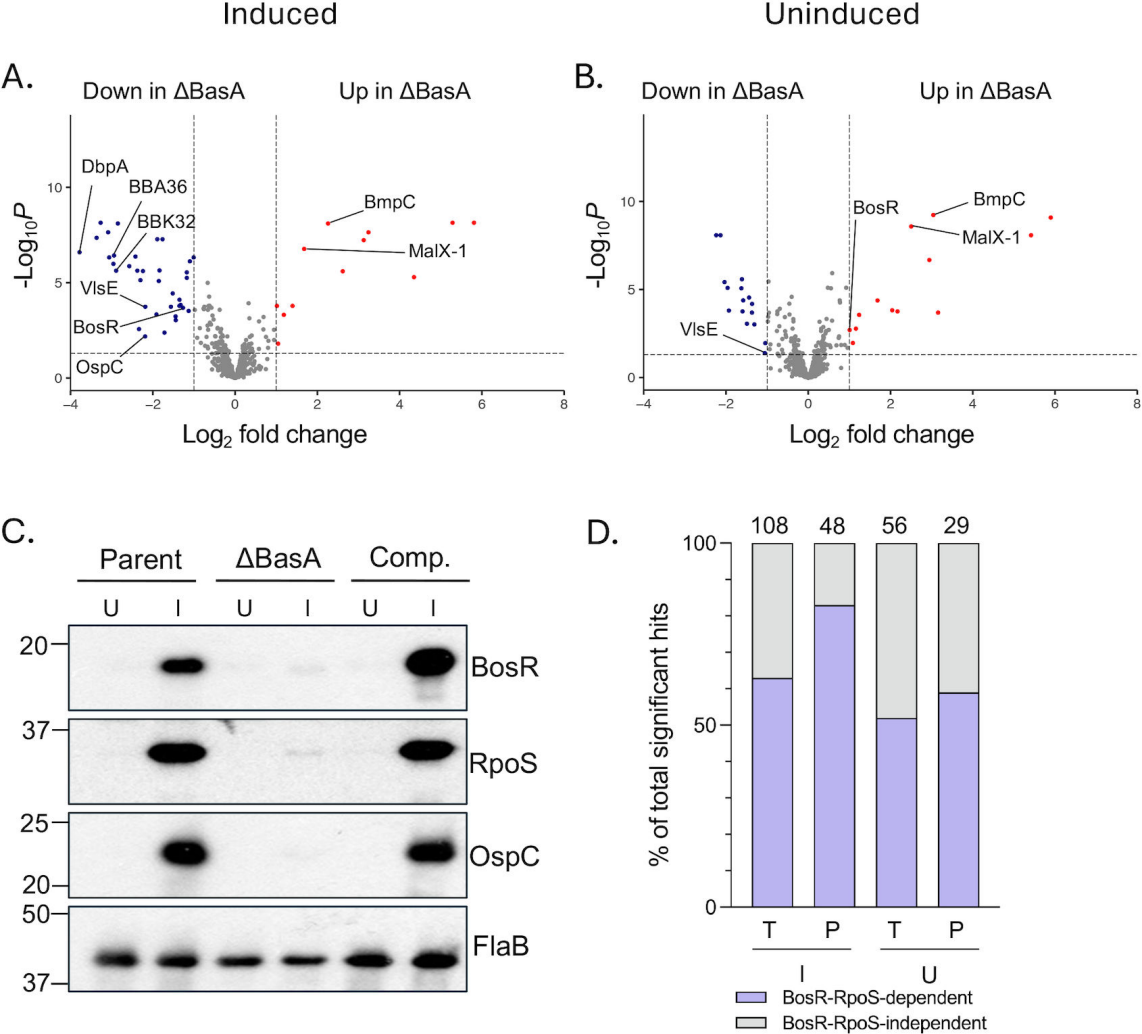
Deletion of BasA alters *B. burgdorferi* proteomic profiles relative to the parent. (**A**) Volcano plot depicting differential protein production in the ΔBasA mutant relative to the parent under induced conditions. Log_2_ fold change in protein abundance is plotted on the x-axis and the FDR corrected *P*-value is plotted on the y-axis. Dots on the volcano plot represent individual proteins. Proteins that met the criteria for statistical significance (FDR-corrected *P*-value < 0.05 with an absolute log_2_ fold change >1) are labeled in blue and red for downregulated and upregulated, respectively. (**B**) Same as panel (**A**) except cells were grown under uninduced conditions. (**C**) Western blot using *B. burgdorferi* protein lysates from the parent, mutant, and complement strains grown under uninduced (“U” = 32˚C, 1% CO_2_, pH 7.6) and induced conditions (“I” = 37˚C, 5% CO_2_, pH 6.8). Lysates were probed with monoclonal antibodies specific for BosR, RpoS, and OspC as well as anti-FlaB as a loading control. (**D**) The percentage of total transcripts “T” and proteins “P” dysregulated by the loss of BasA that are regulated by BosR-RpoS are plotted for induced “I” and uninduced “U” conditions and the total number of dysregulated transcripts or proteins is shown at the top of each bar. The percentage of BosR-RpoS-dependent transcripts or proteins (purple) relative to those that are BosR-RpoS independent (gray) is indicated.

In addition to the transcriptomics, global protein profiles for the same cultures were obtained using Tandem Mass Tags (TMT) mass spectrometry. Proteomic analyses comparing the parent and ΔBasA mutant under inducing conditions revealed 36 downregulated proteins and 12 upregulated proteins in the ΔBasA mutant relative to the parent (Fig. 3A; Table S4). Twenty-nine RpoS-dependent genes dysregulated in the transcriptomics analysis were also significantly dysregulated in the proteomics analysis, including DbpA, OspC, and VlsE. Overall, 89% of downregulated proteins and 69% of upregulated proteins in the ΔBasA mutant relative to the parent are known to be regulated by BosR-RpoS (21, 28). However, unlike in the transcriptomics data, BosR protein production was significantly disrupted in the absence of BasA. This observation, coupled with the similar *bosR* transcript levels between the parent and the ΔBasA mutant, further suggests that BasA functions at a post-transcriptional level to regulate *bosR* transcripts.

Under uninduced growth conditions when BosR and RpoS production are low, the loss of BasA had a subtle effect on global protein dysregulation, with only 16 proteins (Fig. 3B; Table S4) downregulated in the ΔBasA mutant relative to parent (as compared to the 36 proteins that were reduced in the ΔBasA mutant under induced growth conditions [Fig. 3A]). Four proteins, BBF23, BBF24, VlsE, and ErpL, are downregulated in the ΔBasA mutant under both induced and uninduced growth conditions, while 12 proteins are uniquely downregulated in the ΔBasA mutant under uninduced growth conditions. Of the proteins upregulated in the ΔBasA mutant relative to the parent, seven are upregulated in both growth conditions, while five are uniquely upregulated under uninduced growth conditions and four are uniquely upregulated under induced growth conditions. Of note, BosR is upregulated two-fold in the ΔBasA mutant relative to the parent in uninduced conditions (Fig. 3B). This is opposite to what is seen under induced growth conditions but likely reflects the very low-level detection of BosR produced in either the parent or ΔBasA mutant under uninduced growth conditions (Fig. 3C).

Chromosomal complementation of BasA restored 68% of proteins dysregulated by the loss of BasA under induced conditions and 80% in uninduced conditions (Fig. S2C and D). As with the RNA-seq analysis, differences observed between the parent and complement strains can be attributed to proteins on lp36 and cp32-6 plasmids (Table S4). Consistent with the proteomic analysis, Western blotting confirmed the reduction of BosR, RpoS, and OspC in the ΔBasA mutant relative to the parent and complement (Fig. 3C). The loading control, FlaB, is slightly reduced in the ΔBasA mutant relative to the parent and complement lysates. However, this subtle difference does not alter the interpretation that BosR, RpoS, and OspC are significantly reduced in the mutant background (Fig. 3C).

Overall, 66% of transcripts dysregulated by the loss of BasA under induced growth and 48% under uninduced conditions are known to be regulated by the BosR-RpoS regulon (Fig. 3D). Similarly, 83% of proteins dysregulated by the loss of BasA under induced conditions and 59% under uninduced conditions are known to be regulated by BosR-RpoS (Fig. 3D) (21, 28). The dysregulation in the ΔBasA mutant being linked to the BosR-RpoS regulon is striking considering only 10% of the borrelial genome is RpoS-regulated (21). Taken together, the transcriptomics and proteomics results suggest that the regulatory role of BasA functions, in part, at the level of *bosR* post-transcriptional regulation. We hypothesize that BasA promotes the translation of BosR protein, leading to enhanced levels of RpoS and the concomitant production of RpoS-regulated virulence determinants.

### BosR binds directly to BasA in a dose-dependent manner *in vitro*

**Fig 4:**
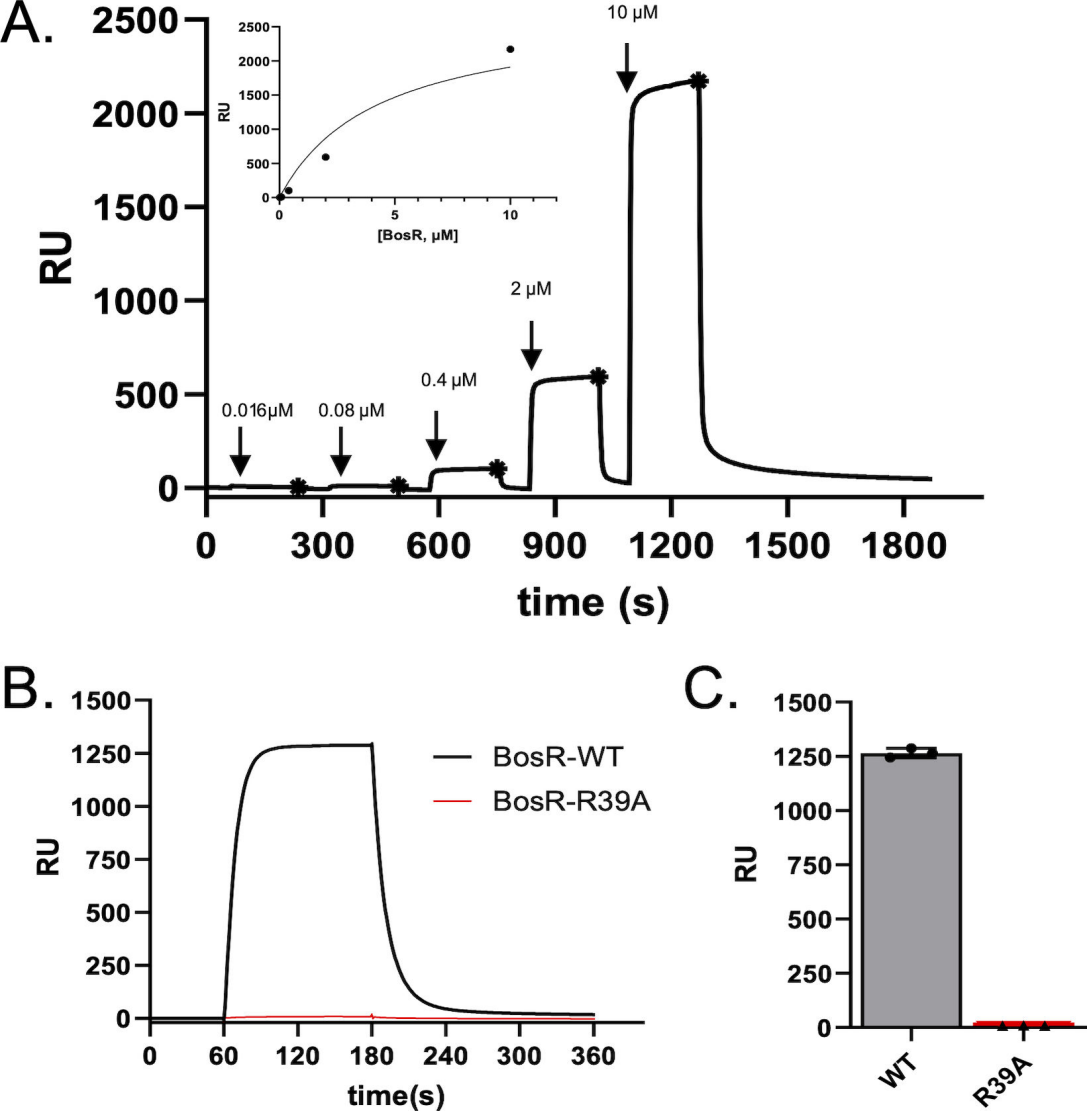
BosR binds BasA *in vitro*. (**A**) Single-cycle SPR binding assays were performed using a fivefold dilution series of BosR-WT (0.016, 0.08, 0.4, 2, and 10 µM) injected over a biotinylated BasA RNA biosensor. Steady-state responses, measured in resonance units (RU), are indicated for each injection with a closed asterisk. A *K*_D_ value of 4.30 µM (S.E. = 0.57 µM) was calculated by fitting the resulting isotherm (inset) to a 1:1 steady-state model of binding. A representative sensorgram is shown. Injections were performed in triplicate. (**B**) A representative sensorgram resulting from an injection of a single concentration (2 µM) of BosR-WT (black) or BosR-R39A (red) over the BasA biosensor is shown. (**C**) Each injection was performed in triplicate, and the steady-state binding responses in RU are plotted. Error bars represent the standard deviation.

Due to the decreased BosR protein production in the ΔBasA mutant and BosR’s recent characterization as an RNA-binding protein (33) with *in vitro* chaperone activity (34), we were interested in testing whether a direct interaction occurs between BosR and BasA using surface plasmon resonance (SPR). Previous literature has indicated that arginine 39 (R39) in BosR is critical to its role in DNA binding (29, 31). Given the putative role for this residue in mediating direct interaction with DNA, we used a BasA biosensor to evaluate the binding activity of recombinant wild-type BosR protein versus a BosR mutant protein with arginine 39 replaced with alanine (BosR-R39A). A BasA biosensor was generated by capturing a commercially synthesized biotinylated form of purified BasA on a streptavidin-derivatized SPR sensor chip. A concentration series of purified BosR was then injected over the BasA biosensor using a single-cycle injection strategy (55). Dose-dependent binding was observed, and fitting of the data to a simple 1:1 steady-state affinity model yielded a calculated equilibrium dissociation constant of *K*_D_ = 4.30 ± 0.57 µM (S.E.) (Fig. 4A) (29, 31). No detectable binding signal was observed when BosR-R39A was injected at 2 µM (Fig. 4B and C). Given the robust response by the wild-type BosR protein at this concentration (Fig. 4B and C), this result indicates that BosR binds directly to BasA *in vitro,* and R39 is a critical residue that promotes the affinity of this protein-RNA interaction (29, 31).

### The loss of BasA significantly attenuates *B. burgdorferi* mammalian infectivity

**Fig 5:**
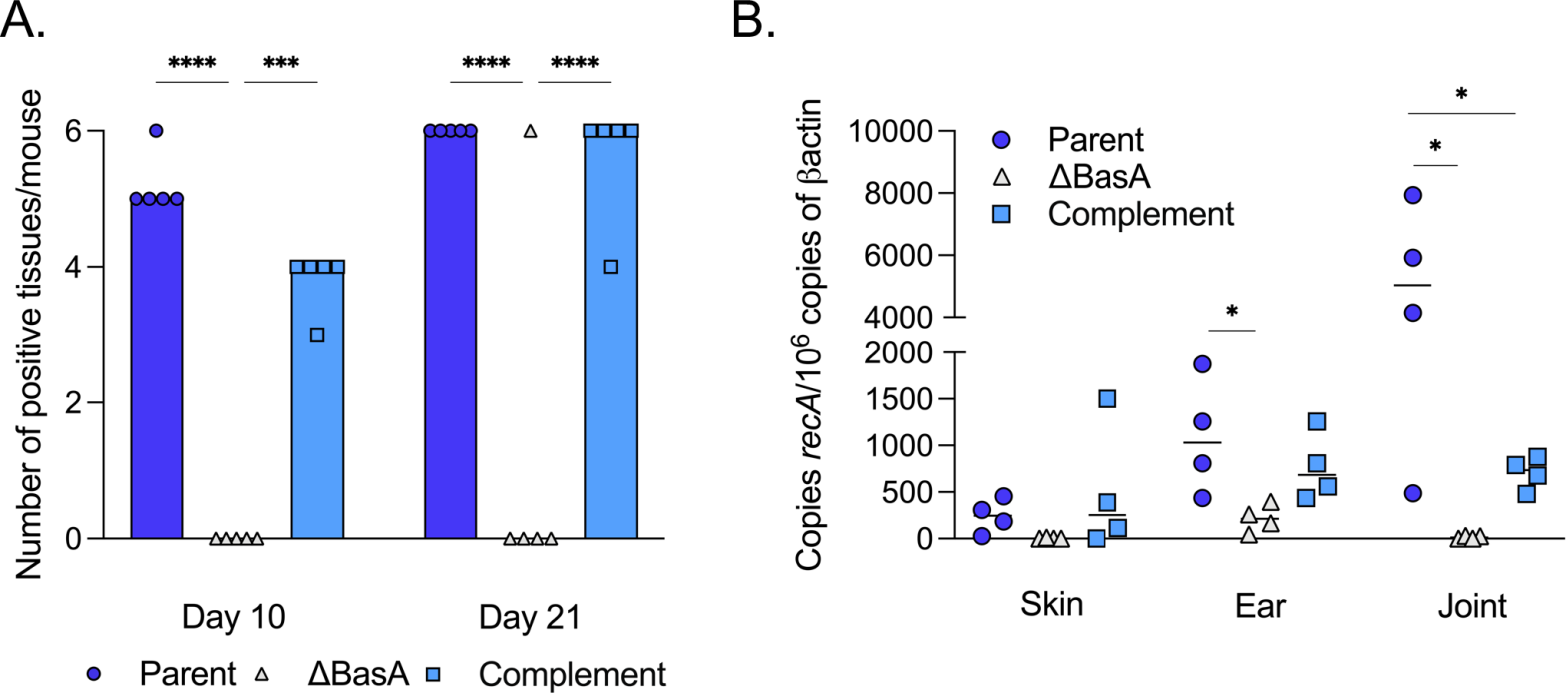
Loss of BasA attenuates *B. burgdorferi* infectivity. Mice were infected with 10^5^ of the *B. burgdorferi* parent (dark blue circles), ΔBasA mutant (gray triangles), or complement (light blue squares) and sacrificed after 10 or 21 days. (**A**) Skin at inoculation site, ear skin, inguinal lymph node, heart, bladder, and tibiotarsal joint were harvested for *in vitro* cultivation of *B. burgdorferi*. Data points represent individual mice in each experimental group. Bars on the histogram represent the median number of positive tissues per mouse. *** *P* < 0.001, **** *P* < 0.0001, determined using a two-way ANOVA with a Tukey’s comparison. (**B**) Quantitative PCR was used to quantify borrelial DNA relative to murine DNA in tissues harvested from mice infected with the parent, ΔBasA mutant, or complement strains. Genomic copies of *B. burgdorferi recA* per 10^6^ copies of murine β-actin are plotted for skin near the inoculation site, ear skin, and the tibiotarsal joint. Data points represent DNA from one individual mouse analyzed by qPCR in triplicate and averaged. The horizontal line depicts the median. *P < 0.05, determined using a one-way ANOVA with a Tukey’s comparison.

Because the ΔBasA mutant produced less key virulence-associated proteins, including BosR, RpoS, and OspC, we evaluated the infectivity potential of the ΔBasA mutants in mice by needle inoculation. C3H/HeN mice were intradermally inoculated on the abdomen with 1 × 10^5^ of either the parent, ΔBasA mutant, or complement strains (*n* = 10 mice per strain). Five mice per strain were sacrificed separately on day 10 and day 21, and tissues were scored qualitatively for outgrowth in culture and quantitatively by qPCR (Fig. 5; Table 1). After 10 days, the only tissue that was not colonized in mice infected with the parent strain was the ear, which represents a distal dissemination tissue for borrelial infection from the abdominal inoculation site (56). Similarly, the complement strain had not fully colonized the ear, heart, or joint tissue on day 10, while no tissues from mice infected with the ΔBasA mutant were culture positive at this time point. After 21 days, tissues from a single mouse infected with the ΔBasA mutant were culture positive for *B. burgdorferi*; the remaining four mice infected with the ΔBasA mutant remained negative (Fig. 5A; Table 1). All tissues from mice infected with the parent and 93.3% of the tissues from complement-infected mice were culture positive at the 21-day time point (Fig. 5A; Table 1). The reduced bacterial load associated with the complement in the joint may be due to the absence of SR0734 (Fig. 1A). However, despite this difference, it is important to note that the BasA complement mimics the parent in the induction of BosR, RpoS, and OspC and overall infectivity (Fig. 3C and 5A). Furthermore, quantification of *B. burgdorferi* genomes from infected tissues revealed significantly less borrelial burden in both the joint and ear by the ΔBasA strain relative to the parent, indicating that BasA is required for disseminated infection (Fig. 5B). Taken together, these results indicate that BasA is required for optimal infection by *B. burgdorferi*.

**TABLE 1:** Infectivity of the ΔBasA mutant strain relative to its parent and genetic complement^*a*^

	Inoculation skin site	Ear	Lymph node	Heart	Bladder	Joint	Total
Day 10							
Parent	5/5	1/5	5/5	5/5	5/5	5/5	26/30
ΔBasA	0/5	0/5	0/5	0/5	0/5	0/5	0/30
Complement	5/5	1/5	5/5	0/5	5/5	3/5	19/30
Day 21							
Parent	5/5	5/5	5/5	5/5	5/5	5/5	30/30
ΔBasA	1/5	1/5	1/5	1/5	1/5	1/5	6/30
Complement	5/5	4/5	5/5	4/5	5/5	5/5	28/30

^*a*^ Number of culture-positive tissues over total number of tissues scored are shown at day 10 and day 21 post-infection for mice infected with 10^5^ of the parent *B. burgdorferi* strain 5A4 NP1, the ΔBasA mutant, or the BasA complement.

## DISCUSSION

**Fig 6:**
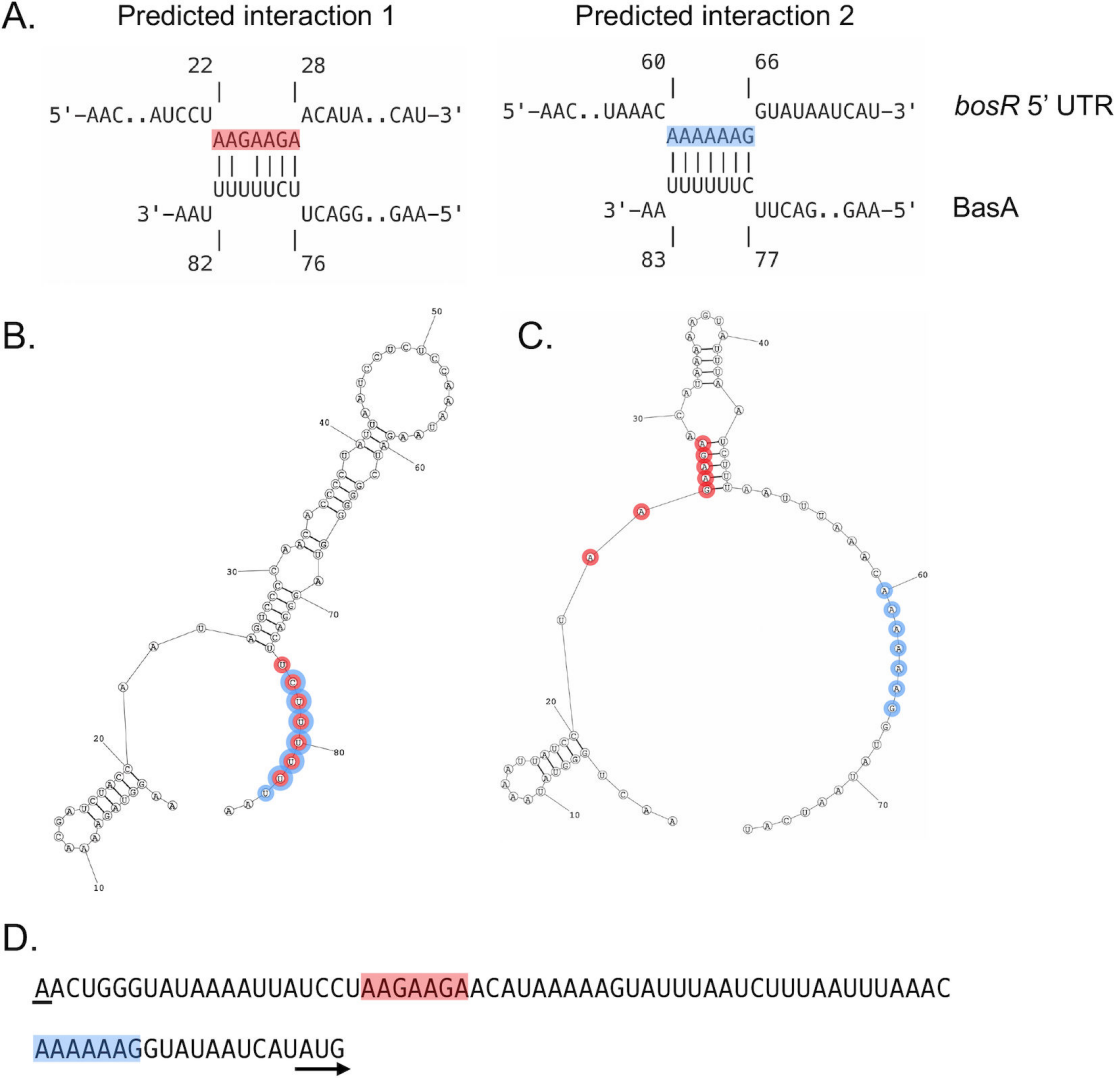
Predicted interactions of BasA with the *bosR* 5′ UTR. (**A**) IntaRNA (Freiburg RNA Tools) predictions of BasA (bottom) and the *bosR* 5′ UTR (top) are shown. The red and blue highlighted regions refer to the *bosR* 5′ UTR sequences that are predicted to interact with BasA. (**B**) The predicted secondary structure of BasA (RNAStructure) and (**C**) for the *bosR* 5′ UTR are shown (RNAStructure) (72). The red and blue highlighted region of BasA indicates the interactive region that is predicted to bind two regions of the *bosR* 5′ UTR and corresponds to the highlighted regions in (**A**). The two sequences in the *bosR* 5′ UTR are also highlighted in red and blue in (**C**, **D**). The *bosR* 5′ UTR sequence is shown with the predicted interactions from panel (**A**) highlighted in red and blue. The underline indicates the transcriptional start site, and the *bosR* start codon is designated with an arrow.

**Fig 7:**
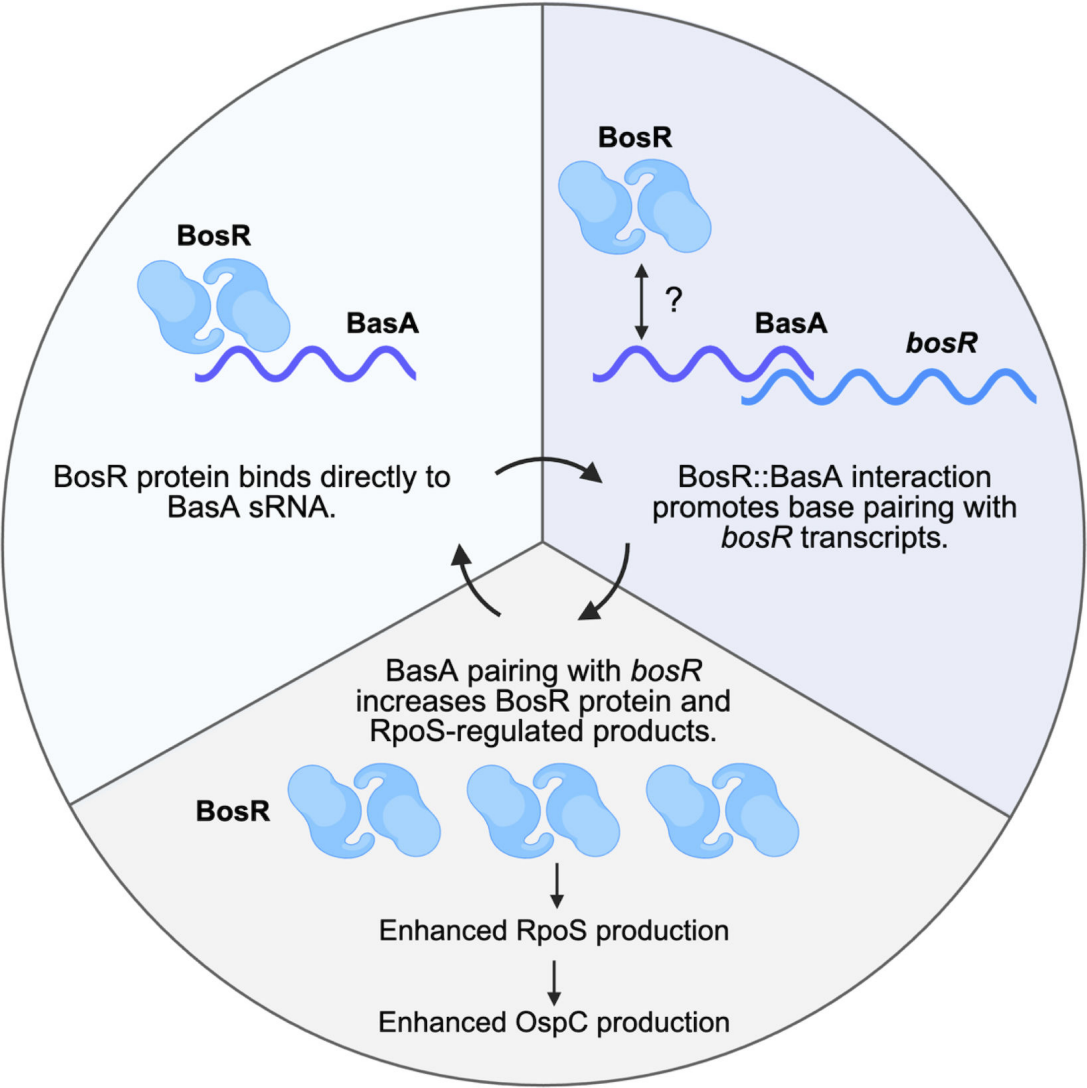
Proposed model for BasA post-transcriptional regulation of *bosR*. The *in vitro* binding data suggest BosR protein binds directly to BasA sRNA (top left). We hypothesize that this interaction could promote BasA base pairing with target transcripts, likely including *bosR* (top right). BosR could form a complex with BasA and *bosR*, or BosR could release from BasA after BasA pairs with *bosR*. BasA pairing with *bosR* increases BosR protein production and downstream RpoS-regulated products like OspC (bottom). Created in BioRender. Shapiro, B. (2026) https://BioRender.com/x1i19nr.

*Borreliella burgdorferi* is known to modulate its gene expression patterns dramatically during a blood meal as the bacterium moves from the *Ixodes* tick midgut to mammals (3, 7–10). Some of the protein regulators responsible for these shifts in gene expression are known, namely BosR and RpoS, as well as RpoN and Rrp2 (18, 20–22, 25, 27, 28, 57–61). However, the signals at the molecular level that promote increased production of these regulatory proteins are not well characterized (9, 18, 26, 30, 33, 38, 62–71). Prior studies from our group and others have found that BosR is required for successful mammalian infection (23, 25, 27). Our group previously demonstrated that *bosR* gene expression does not change under microaerophilic or anaerobic conditions or when CO_2_ levels are altered; however, BosR protein levels do increase when borrelial cells are grown anaerobically or with higher levels of CO_2_ (5% CO_2_) (26). This observation suggests that *bosR* transcripts are subject to post-transcriptional regulation (26). Historically, the regulatory activity of BosR was attributed exclusively to its DNA-binding activity and subsequent alteration of target gene transcription (23, 25, 27, 29, 30, 66–70). Recently, BosR was found to have RNA-binding activity *in vivo* and can act as an RNA chaperone to anneal two unstructured synthetic RNA molecules *in vitro* (33, 34). These results indicate that BosR could function as an RNA chaperone in *B. burgdorferi*, perhaps as an RNA matchmaker, binding to small, non-coding RNAs (sRNAs) and/or mRNAs (33, 34) to modulate translation. Herein, we demonstrate that BosR binds *in vitro* to the sRNA we designate as BasA. We determined that BasA is required for optimal production of BosR and the downstream regulatory protein RpoS, as well as RpoS-regulated genes and their respective gene products. We also observed a significant reduction of borrelial colonization in mice infected with strains lacking BasA (Fig. 5), consistent with decreased BosR and RpoS production, which phenocopies a *bosR* mutant (25, 27). We hypothesize that BasA interacts with the *bosR* 5′ untranslated region (UTR) and complexes with existing BosR protein to promote optimal BosR protein production (Fig. 6 and 7). The potential role of BasA in *B. burgdorferi* post-transcriptional gene regulation of *bosR* and other transcripts represents an additional tool for borrelial cells to dynamically regulate infectivity-associated protein production in response to environmental changes.

Given the unchanged *bosR* transcript levels and reduction of BosR protein in the absence of BasA (Fig. 2 and 3), BasA either stimulates translation of BosR or inhibits BosR degradation directly or indirectly (71). The simplest model posits that the BosR::BasA complex post-transcriptionally regulates *bosR* transcripts, promoting optimal levels of BosR protein (Fig. 6 and 7). In the absence of BasA, the *bosR* transcript may be inefficiently translated, or the BosR protein may be subjected to degradation, resulting in the reduction of BosR protein and concomitant reduction of RpoS and decreased RpoS-activated products. Specifically, OspC and other RpoS-regulated gene products, such as DbpA and BBK32, were reduced in the ΔBasA mutant (Fig. 3; Table S4), consistent with the proposed model (Fig. 7). The prior post-transcriptional regulation we observed under induced conditions for *bosR,* whereby *bosR* transcripts remain unchanged but BosR protein levels increase (26), suggests that BasA may be contributing to the post-transcriptional regulation of the *bosR* transcript. This hypothesis is further supported by two potential recognition sequences between BasA and the *bosR* 5′ UTR, which could then alter the translation of the *bosR* transcript (Fig. 6). Further analysis is required to demonstrate a direct link of BasA to the *bosR* transcript and to determine the role that BosR might play in this process.

The moderate *in vitro* binding affinity of BosR to BasA (Fig. 4B), as well as *rpoS* mRNA (33), may be inherent to its function as an RNA-binding protein or due to the constraints of *in vitro* analyses. Protein RNA-binding activity could be transient, such that subsequent recycling of this interaction might mediate additional adaptive responses (34). It is possible that other factors may be required for optimal binding *in vivo*, including oligomerization, metal coordination, target transcripts, and other borrelial components that might stabilize BosR::BasA interactions (34). To address additional BosR::BasA interactions, we are currently characterizing the ability of BosR to bind model RNAs using various redox permutations that may alter its activity. This additional experimentation will begin to address the underlying molecular mechanisms that govern RNA interactions by BosR.

When we tested the BasA deletion in the murine experimental model of Lyme borreliosis, *B. burgdorferi* infection was significantly reduced (Fig. 5; Table 1). The attenuation observed in the BasA background was predicted, given the reduction in BosR, RpoS, and OspC (Fig. 3C). Notably, complementation with native BasA restored antigen production and infectivity (Fig. 2 and 3; Table 1). However, one of the five mice was culture positive when infected with 10^5^ ΔBasA *B. burgdorferi*. Subsequent analysis confirmed the presence of the BasA deletion, indicating that, in some instances, the absence of BasA can be overcome (Fig. S3). For infection to occur in the ΔBasA isolate, enough BosR would need to be made in a subset of cells to then produce enough RpoS to establish infection. Alternatively, the ΔBasA population in this mouse could have acquired a compensatory mutation or change in epigenetic regulation that allowed it to overcome the loss of BasA. Furthermore, our results are striking considering an lp17 mutant missing *bbd16* through *bbd25*—that includes BasA—had a reduced but more subtle infectivity phenotype in a separate independent study (73). Nevertheless, like the work shown here, this prior work demonstrated a reduced infection phenotype indicative of a deficiency in disseminated borrelial infection (73).

One limitation of this work is the absence of any direct link to the BosR::BasA complex *in vivo* and with BasA-targeted transcripts. Further experimentation, analyzing BosR::BasA *in vivo,* together with BasA::transcript ligation, is needed to provide a comprehensive catalog of mRNA targeted by BasA. The model we propose here predicts that BosR could facilitate BasA’s interaction with the *bosR* 5′ UTR, resulting in optimal translation to produce BosR. Interestingly, an additional sRNA, designated SR0546, overlaps with the *bosR* 5′ UTR (46). How SR0546 interacts with BasA and the role this interaction plays in the regulatory response seen for BosR production remains to be determined. Alternatively, other borrelial components and/or cofactors may be required to stabilize the *bosR* mRNA and enhance its protein levels. This type of complex may be key to evaluate how BasA functions, together with BosR, to promote translation of *bosR* transcripts and perhaps other transcripts as well. Future RNA ligation experiments (74, 75) designed to characterize the transcripts that bind to BasA should identify additional targets as well as other candidates that can be evaluated in this context.

## MATERIALS AND METHODS

### Bacterial strains and culture conditions

Plasmids and bacterial strains used in this study are described in Table S1. *E. coli* strains were grown at 37°C in Lysogeny Broth (LB) broth or low salt Lennox LB broth supplemented with antibiotics at the following concentrations: kanamycin, 50 μg/mL; gentamicin, 5 μg/mL; spectinomycin, 50 μg/mL; or hygromycin, 20 μg/mL. *B. burgdorferi* strains were grown in BSK-II media (76) supplemented with normal rabbit serum (NRS) (Pel-Freez Biologicals) in uninduced conditions defined as 32°C, pH 7.6, and 1% CO_2_ or BosR-RpoS regulon induced conditions at 37°C, pH 6.8, and 5% CO_2_ (47, 77). Complete BSK-II media (with NRS) was supplemented with antibiotics at the following concentrations: kanamycin, 300 μg/mL; gentamicin, 50 μg/mL; or hygromycin, 300–400 μg/mL.

### Genetic inactivation of *B. burgdorferi* BasA

BasA was inactivated via homologous recombination by replacing the intergenic sRNA with P*_flgB_-*gent^R^ antibiotic cassette (78). Sequences flanking BasA, approximately 1.5 kb in length, were amplified from *B. burgdorferi* genomic DNA using PCR with PrimeSTAR GXL polymerase (Takara). The P*_flgB_*-gent^R^ cassette was amplified from pBSV2G (78). The primers used for PCR and details regarding the amplified products are shown in Table S1. The three fragments with overlapping sequences were assembled using NEBuilder HiFi DNA Assembly Master Mix (NEB) prior to ligation with TOPO Blunt II (Thermo Scientific). The resulting construct was designated pWE100 (Table S1).

To generate the BasA complement construct, BasA and 82 bp upstream of BasA were restored into the *B. burgdorferi* chromosome between *bb0445* and *bb0446* via homologous recombination (49). Chromosomal regions flanking the insertion site were amplified using primers listed in Table S1. The P*_flgB_*-hyg^R^ cassette and 114 bp upstream of P*_flgB_* were amplified from pBSV2H (Table S1) (79). BasA was amplified from lp17 using purified *B. burgdorferi* genomic DNA as template (Table S1). The four PCR fragments with overlapping sequences were assembled following the same protocol used to generate pWE100. The resulting construct was designated pWE135 (Table S1).

### Transformation of *B. burgdorferi*

Prior to transformation, pWE100 and pWE135 were linearized by digestion with *Bam*HI (NEB). Competent *B. burgdorferi* cells were prepared and electroporated with linearized plasmid DNA into cells as previously described (80–84). Putative transformants were screened by PCR using primers listed in Table S1, and for endogenous plasmid content as previously described (85).

### SDS-PAGE and immunoblotting

*B. burgdorferi* protein lysates were resolved on SDS-12.5% polyacrylamide gels, transferred to PVDF membranes, and blocked in 5% non-fat milk (29, 77, 86). Monoclonal antibodies were used as primary antibodies at the following dilutions: anti-BosR 1:2,500 (contracted to and produced by ProMab), anti-RpoS 1:1,000 (ProMab), anti-OspC 1:250,000 (ProMab), and anti-FlaB 1:20,000 (US Biological). Anti-mouse Ig secondary antibody conjugated to horseradish peroxidase (HRP) (Life Tech Stockroom) was used at a 1:10,000 dilution to detect membrane-bound immune complexes following incubation with the Western Lightning Plus-ECL system (Revvity Inc.). Blot images were obtained using autoradiological film.

### RNA sequencing (RNA-seq)

Three independent 150 mL cultures of the *B. burgdorferi* parent strain 5A4 NP1, the ΔBasA mutant, and the BasA chromosomal complement were grown to 5–8 × 10^7^ cells/mL in both uninduced and induced conditions. RNA was isolated and DNase treated as described (87, 88). RNA quality was assessed by resolution on a 2% agarose gel and quantified using Nanodrop (Thermo Scientific). Additionally, RNA was used as a template in a PCR with primers to *flaB* to assess the removal of contaminating DNA. 2 μg RNA per strain in triplicate was sent to SeqCenter (Pittsburgh, PA) where an additional DNase (Invitrogen) treatment was performed followed by library preparation using the Total RNA Prep Ligation with Ribo-Zero Plus kit (Illumina) and 10 bp unique dual indices (UDI). rRNA depletion was performed using custom probes designed for borrelial rRNAs available at SeqCenter. Libraries were sequenced on NovaSeq X Plus, producing paired-end 150 bp reads. On average, each sample generated 16.9 million reads. Bcl-convert (v4.2.4) was used for demultiplexing, quality control, and adapter trimming after sequencing. Trimmed reads were mapped to *B. burgdorferi* B31 reference genome (GCF_000008685.2) in CLC Genomics Workbench v25.0.1, allowing only one hit per read using a minimum length fraction of 0.9 and a minimum similarity fraction of 0.9. Significant differential expression between strains grown under uninduced or induced conditions was determined in CLC Genomics Workbench using the “Differential Expression in Two Groups” tool. Genes were considered significantly differentially expressed if the FDR-corrected *P*-value was <0.05, the absolute log_2_ fold change was >1, and the mean number of reads per group was >15 (0.00009% of total reads per sample) (Table S3). Volcano plots were generated in CLC Genomics Workbench. The RNA-seq raw data are available at NCBI BioProject via accession number GSE309982.

### RNA for conventional RT-PCR and RT-qPCR

Purified RNA samples from the same three independent cultures of parent strain *B. burgdorferi* 5A4 NP1, the ΔBasA mutant, and the BasA chromosomal complement were used for RT-PCR and RT-qPCR. The RNA was DNase treated and converted to cDNA as previously described (87, 88). PCRs were run with 100 ng cDNA as template using PrimeSTAR GXL polymerase (Takara) and primers listed in Table S1. RT-qPCRs amplified 10 ng cDNA with Perfecta SYBR Green FastMix (Quantabio). For quantitative detection, transcript levels of selected borrelial transcripts were normalized to the constitutively expressed *flaB* gene. *B. burgdorferi* targets were amplified using primers described in Table S1.

### Tandem mass tags mass spectrometry

The same cultures grown for RNA-seq were also analyzed by tandem mass tag (TMT) mass spectrometry, and protein samples were prepared as previously described (47). TMT MS was performed by the University of Texas Southwestern proteomics core. 1.2 mg protein per strain in triplicate was used for TMT analysis. Protein extracts were reduced, alkylated, and proteolytically digested overnight before being labeled with the TMT reagents in a 6-plex experiment, followed by fractionation and clean up. Samples were then analyzed by Orbitrap LC-MS/MS. Results were analyzed using Proteome Discoverer 3.0. A total of 851 *B. burgdorferi* proteins were detected in cells grown under induced conditions based on False Discovery Rates of <5%. Significant differential production between groups was determined using the R package “MSstatsTMT” (89). The “msstats” method was used for protein summarization, and missing data were imputed. We required at least two unique peptides for protein identification. Protein abundances were compared across conditions using a linear mixed-effects model with a moderated t-statistic. Proteins were considered differentially produced if the FDR-corrected *P*-value was <0.05, and the absolute log_2_ fold change was >1. A similar analysis was done for the complement relative to both the parent and mutant under induced conditions. Volcano plots were generated in R using the “EnhancedVolcano” package (https://github.com/kevinblighe/EnhancedVolcano). The TMT raw data were deposited to MassIVE using Proteomic Xchange Consortium with accession number MSV000099829 and are available for download at https://massive.ucsd.edu/ProteoSAFe/dataset.jsp?task=d740dccdcbd545b5adb43eb27d4598c7.

### Production of recombinant proteins

DNA fragments encoding wild-type BosR from *B. burgdorferi* (UNIPROT: BB_0647) and BosR containing a point mutation (i.e., BosR-R39A) were codon-optimized for *E. coli* and synthesized using Integrated DNA Technologies gBlock Gene Fragment service, flanked with 5′ *Bam*HI and 3′ *Not*I sequences, and were cloned into the pT7HMT vector (90). Sequence-confirmed plasmids were transformed into BL21(DE3) cells for protein production following the general protocol previously described (55). Following induction and cell lysis, the soluble fraction was suspended in a buffer containing 10 mM Tris pH 8.0, 10 mM imidazole, and 500 mM NaCl (native binding buffer), passed gradually over an Ni-NTA column previously equilibrated with five column volumes of native binding buffer, washed with 10 column volumes of native binding buffer, and eluted with a buffer containing 10 mM Tris pH 8.0, 500 mM Imidazole, and 500 mM NaCl. The affinity tag was removed by the addition of His-tagged tobacco etch virus protease (His-TEV), 1 mM DTT, and 1 mM EDTA. The reaction was dialyzed overnight at room temperature into native binding buffer. The sample was then passed over a Ni-NTA column, the flowthrough collected, and further purified by size-exclusion chromatography using a HiLoad 26/600 Superdex 75 PG gel filtration column (Cytiva) in a buffer of 10 mM HEPES pH 7.3, 140 mM NaCl. Fractions containing pure BosR protein, as judged by SDS-PAGE, were pooled, concentrated, and stored at −80°C until use.

### Surface plasmon resonance

All SPR experiments were performed on SAD 200M (Streptavidin modified carboxymethyl dextran) sensor chips manufactured by Xantec bioanalytics. SAD chips were preconditioned with three 60 s injections of 1M NaCl and 50 mM NaOH, followed by equilibration until baseline stability was achieved. Commercially synthesized BasA RNA, modified with a 5′-biotin, was obtained from Horizon Discovery Biosciences and resuspended in 10 mM HEPES, pH 7.4, at a stock concentration of 100 µM. Just prior to immobilization, RNA was refolded by heating to 80°C for 2 min and then passively cooling to 25°C. Biotinylated (5′) BasA RNA was captured on the SAD chip at a final immobilization level of 3,115 RU. A fivefold dilution series of BosR-WT was prepared (10–0.016 µM) and injected over the RNA biosensor using a single-cycle injection protocol with an association phase of three min and a flow rate of 30 µL/min. Dissociation for the final injection was monitored for 10 min, at which point baseline response was achieved. Reference-corrected sensorgrams were fit to a 1:1 steady-state affinity binding model constrained by the maximal theoretical experimental binding response based on the molecular weight of the BosR analyte and immobilization of the BasA ligand using Biacore T200 Evaluation Software. Single concentration (2 µM) injections for BosR-WT and BosR-R39A were performed in a similar manner. In all cases, injections were performed in triplicate.

### Infectivity studies

Animal procedures were reviewed and approved by the Texas A&M University Institutional Animal Care and Use Committee (IACUC protocol 2022-0309) and are consistent with the guidelines of the Association for Assessment and Accreditation of Laboratory Animal Care (AAALAC). C3H/HeN (Charles River) mice were intradermally inoculated with 10^5^ organisms of the *B. burgdorferi* parent strain 5A4 NP1, the BasA mutant, or the BasA chromosomal complement. Each group consisted of 10 mice. After 10 and 21 days, five mice from each group were sacrificed, and abdominal skin (inoculation site), ear, inguinal lymph node, heart, bladder, and tibiotarsal joint tissues were collected from each mouse aseptically and cultured in complete BSK-II media under appropriate antibiotic selection (Table S1). Additional abdominal skin (inoculation site), ear, and tibiotarsal joint tissues were collected from each mouse for qPCR analysis of the mice sacrificed on day 21 (see below).

### qPCR analysis

Total DNA was isolated from abdominal skin (inoculation site), ear skin, and tibiotarsal joint tissue from each mouse and used in qPCR to amplify borrelial and mouse targets with primers listed in Table S1 as previously described (47, 86, 91, 92).

### *In silico* prediction of BasA target and secondary structure

The *bosR* 5′ UTR sequence and the BasA sequence were subjected to IntaRNA (Freiburg RNA Tools) binding predictions (72, 93–96). RNAStructure fold was used to predict the BasA and *bosR* 5′ UTR secondary structures (97).

### Statistical analysis

A one-way ANOVA with a Tukey’s comparison was done for each transcript’s expression measured by RT-qPCR and each tissue DNA amplified from the qPCR analysis in GraphPad Prism version 10.4.2. A two-way ANOVA with a Dunnett’s multiple comparison test was performed for the borrelial *in vitro* growth curves and with a Tukey’s comparison for the borrelial outgrowth in GraphPad Prism.

## SUPPLEMENTAL MATERIAL

Fig. S1 iai.00026-26-s0001.tif Growth kinetics of *B. burgdorferi* strains used.

Fig. S2 iai.00026-26-s0002.tiff Transcript and proteomic comparison of the BasA complement relative to the BasA mutant.

Fig. S3 iai.00026-26-s0003.tiff PCR analysis of BasA mutant that was isolated from the sole infected mouse.

Fig. S4 iai.00026-26-s0004.tif Uncropped RT-PCR, PCR, and Western immunoblots.

Supplemental material iai.00026-26-s0005.docx Supplemental methods; Supplemental figure legends.

Supplemental data iai.00026-26-s0006.xlsx Raw data for Figures 2C, 2D, 5B, and S1.

Table S1 iai.00026-26-s0007.xlsx Strains, plasmids, and oligonucleotides used in this study.

Table S2 iai.00026-26-s0008.xlsx Transcripts with an absolute log2 fold change between various strain comparisons (see tabs).

Table S3 iai.00026-26-s0009.xlsx RNA sequencing mapping statistics.

Table S4 iai.00026-26-s0010.xlsx Proteins with an absolute log2 fold change >1 between different strains (see tabs).

ASM does not own the copyrights to Supplemental Material that may be linked to, or accessed through, an article. The authors have granted ASM a non-exclusive, world-wide license to publish the Supplemental Material files. Please contact the corresponding author directly for reuse.
