# Supplementary figures and images for: The Borreliella burgdorferi BosR-associated small non-coding RNA BasA regulates virulence
Source: Infect Immun. 2026 Mar 10;94(4):e00026-26. doi: 10.1128/iai.00026-26 (PMC13081726; doi:10.1128/iai.00026-26)

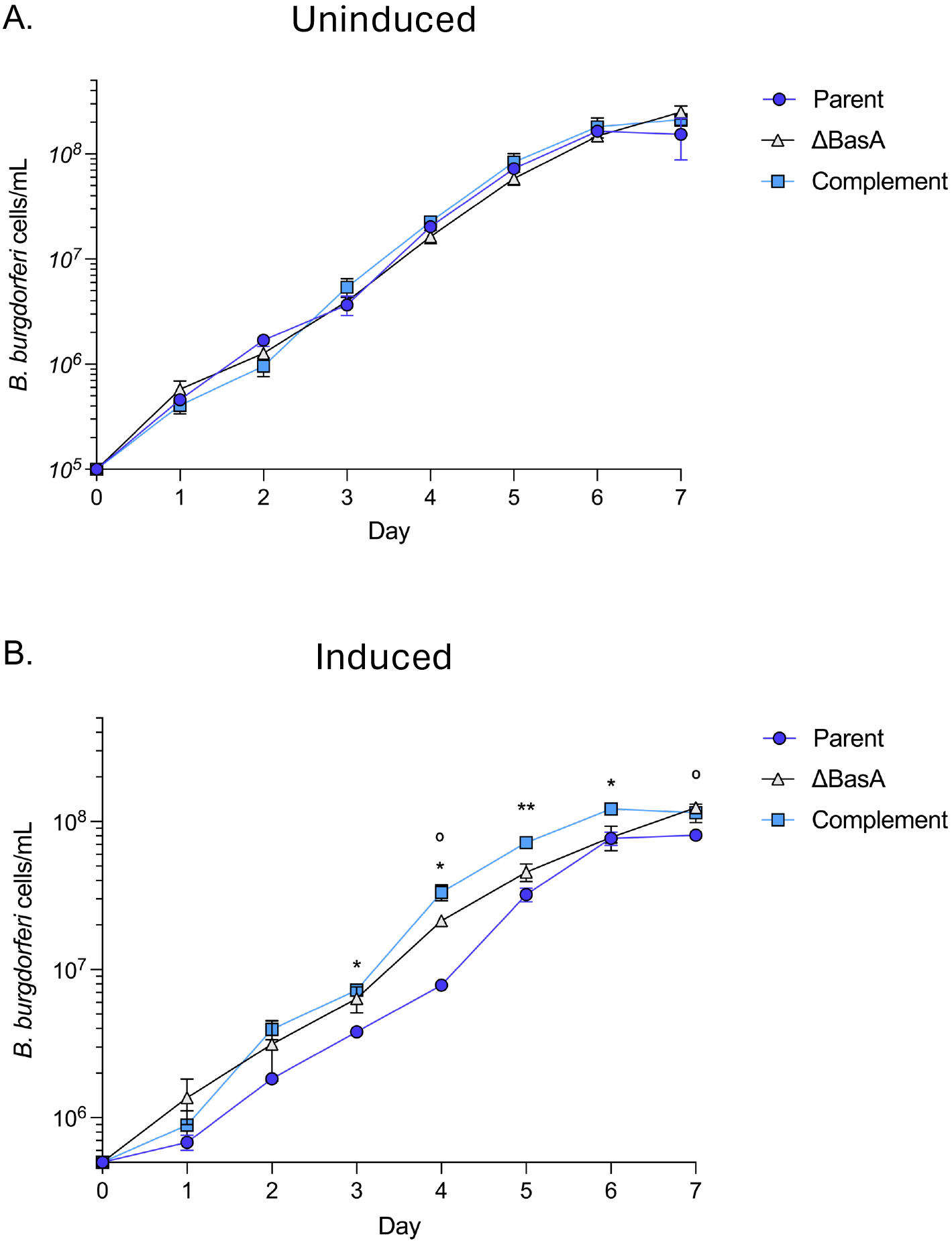

Supplement: Fig. S1 — Growth kinetics of B. burgdorferi strains used. [file iai.00026-26-s0001.tif]

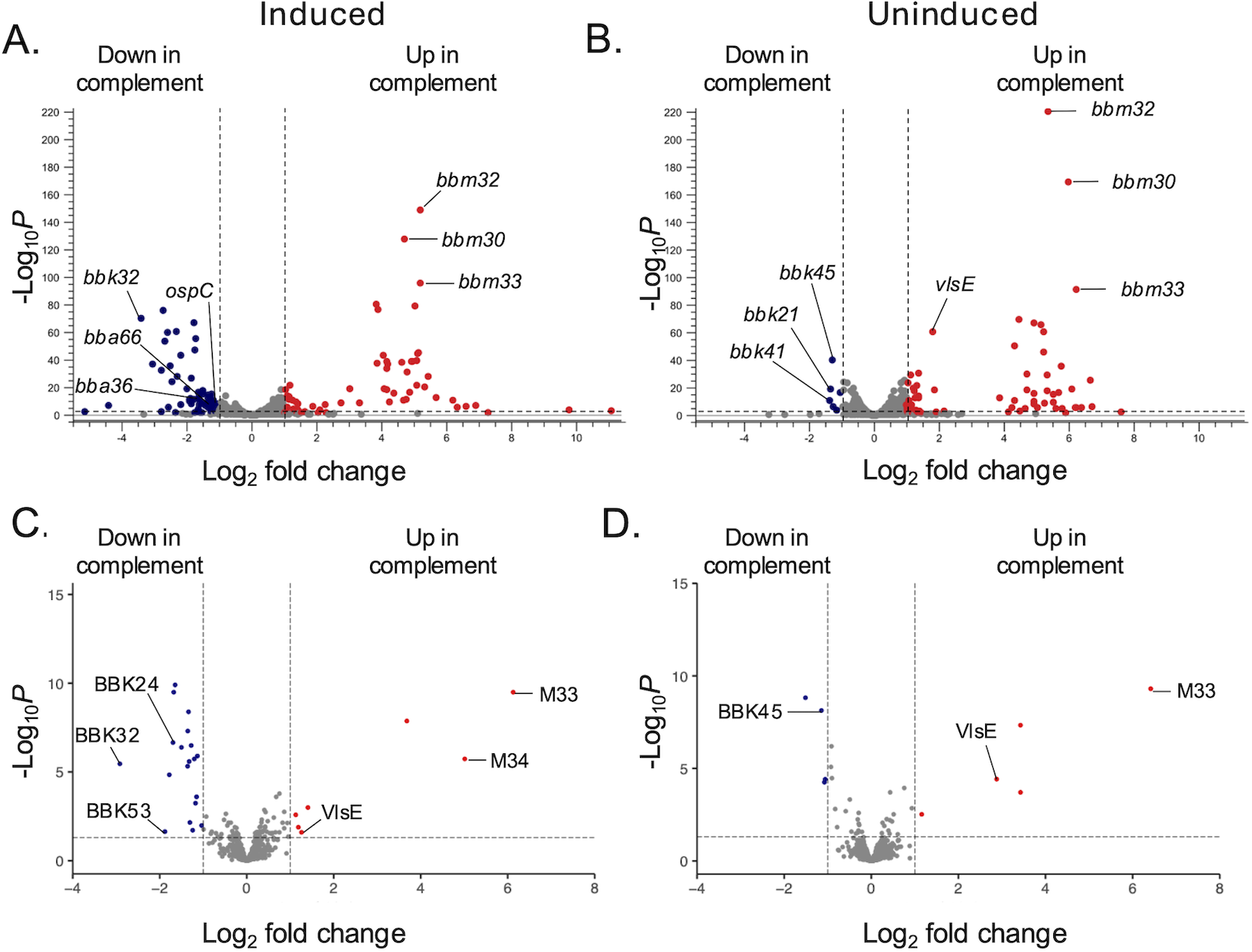

Supplement: Fig. S2 — Transcript and proteomic comparison of the BasA complement relative to the BasA mutant. [file iai.00026-26-s0002.tiff]

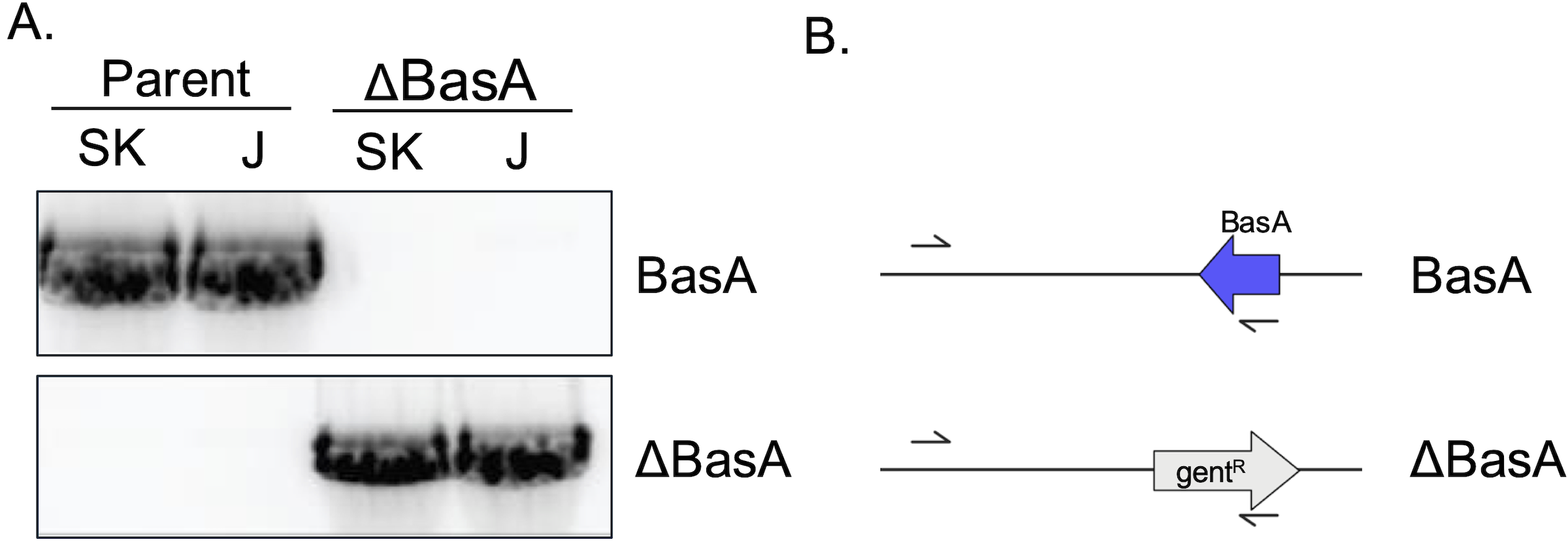

Supplement: Fig. S3 — PCR analysis of BasA mutant that was isolated from the sole infected mouse. [file iai.00026-26-s0003.tiff]

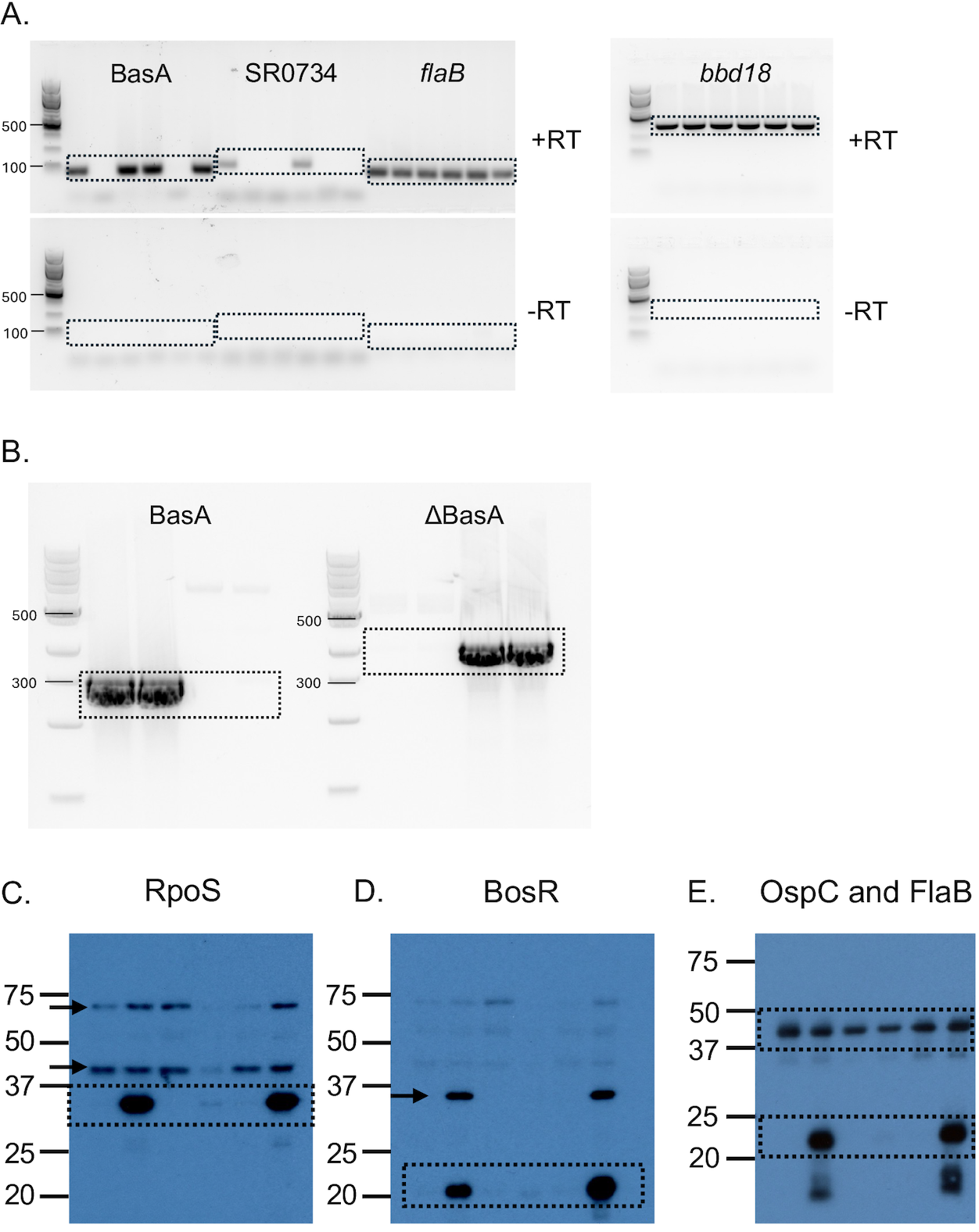

Supplement: Fig. S4 — Uncropped RT-PCR, PCR, and Western immunoblots. [file iai.00026-26-s0004.tif]
