# Supplementary material for: The Borreliella burgdorferi BosR-associated small non-coding RNA BasA regulates virulence
Source: Infect Immun. 2026 Mar 10;94(4):e00026-26. doi: 10.1128/iai.00026-26 (PMC13081726; doi:10.1128/iai.00026-26)
Supplement: Supplemental material — Supplemental methods; Supplemental figure legends. [file iai.00026-26-s0005.docx]

**SUPPLEMENTAL MATERIALS**

**SUPPLEMENTAL METHODS**

**Screening single isolated BasA deletion confers infections.** One of the mice infected for 21 days with the ΔBasA mutant strain at a 10^5^ dose was culture positive for all tissue samples cultivated (**Fig 5** and **Table 1**). To determine whether the infection was the result of a mixed population of *B. burgdorferi*, skin and joint DNA samples that were isolated from the infectious parent and the mutant obtained from the lone infected mouse were subjected to PCR. Primers 1 and 32 were used to amplify the native BasA sequence, and primers 1 and 34 were used to amplify the ΔBasA mutant gentamicin resistance marker (**Table S1**). The resulting amplified products were analyzed by agarose gel electrophoresis (see **Fig. S3**).

**SUPPLEMENTAL FIGURE LEGENDS**

**Supplemental Fig. 1. *In vitro* growth rate of strains used in this study.** Three independent cultures of the parent, the ΔBasA mutant, and the BasA chromosomal complement, were grown in uninduced (32˚C, 1% CO_2_, pH 7.6) indicated in panel **A** and induced conditions (37˚C, 5% CO_2_, pH 6.8) shown in panel **B**. Cell density was quantified by dark field microscopy every 24 hours for 7 days. Significance between the parent and complement is denoted as ** p* < 0.05*, ** p* < 0.01 and between the parent and mutant as *° p* < 0.05 determined using a two-way ANOVA with a Dunnett’s multiple comparison test*.*

**Supplemental Fig. 2. Chromosomal complementation of BasA restores disrupted BosR and RpoS-regulated transcripts and proteins.** The parent and BasA chromosomal complement were grown in biological triplicate under induced and uninduced conditions and subjected to RNA-seq or TMT analysis. The volcano plots depict in panel **A** the differential transcripts in induced and **B** uninduced conditions for the BasA chromosomal complement relative to the parent strain. Differential proteins are depicted in panel **C** in induced conditions and **D** uninduced conditions. Log_2_ fold change in transcript or protein abundance is plotted on the x-axis, and the FDR p-value is plotted on the y-axis in panels **A** and **B**, respectively. Dots on the volcano plot represent individual genes or proteins. Transcripts and proteins that met the criteria for statistical significance (FDR-corrected p-value <0.05 with an absolute log_2_ fold change >1) are labeled in blue or red for up or down-regulated, respectively. Corresponding transcript and protein data is shown in Supplemental Table 4 and Supplemental Table 5, respectively.

**Supplemental Fig. 3. Confirmation that the single murine-isolated BasA** **mutant confers infection.** *B. burgdorferi* were isolated from *in vitro* cultivated murine skin (SK) and joint (J) samples from one of the parent samples and the sole ΔBasA mutant tissue positive samples. DNA was isolated and subjected to PCR using the primers shown on the right. The PCR primer set corresponding to the native BasA sequence is indicated as “BasA” and the primers that amplify the gentamicin resistance (gent^R^) cassette in the Δ*basA* mutant are indicated as “ΔBasA” as shown in the schematic. Created in BioRender. Shapiro, B. (2026) <https://BioRender.com/z10lwd3>.

**Supplemental Fig. 4. Uncropped qualitative RT-PCR and PCR images from Fig. 1 (A) and Fig. S2 (B), respectively, and uncropped western blot images from Figure 2C.** Dotted lines depict the regions that were cropped. **A.** RT-PCR amplification of BasA, SR0734, *flaB, and bbd18* (top) and RT-PCR amplification of BasA*,* SR0734*, flaB, and bbd18* in the no reverse transcriptase control (bottom). **B.** PCR amplification of BasA (left) and amplification of the ΔBasA mutant (right). **C.** RpoS Western blot shown in Fig. 3C. The top arrow points to the non-specific RpoD band that runs at approximately 70 kDa. The bottom arrow points to the non-specific RpoN band that runs at approximately 45 kDa. **D.** BosR Western blot shown in Fig. 3C. The anti-RpoS blot was stripped and re-probed with anti-BosR. An arrow points to the RpoS band cropped from **Fig. S4C**. **E.** FlaB (top) and OspC (bottom) Western blot shown in Fig. 3C. Monoclonal antibodies to FlaB and OspC were combined and the total blot is shown. Markers in kDa are indicated on the left.

**SUPPLEMENTAL TABLES**

**Supplemental Data 1. Excel spreadsheet with numerical data and statistical values for Figs 2C, 2D, 5B, and S1.** Data for each figure is shown in individual tabs.

**Supplemental Table 1. Strains, plasmids, and oligonucleotides used in this study.**

**Supplemental Table 2. Transcripts with an absolute log_2_ fold change >1 in RNA-seq comparisons.** Nomenclature is based on new and old locus tag designations derived from *B. burgdorferi* B31 RefSeq (NCBI ID: GCF_000008685.2). Transcripts that map to pseudogenes are not included in this table. Transcripts listed met the criteria for statistical significance. Genes associated with BosR-RpoS regulation defined as a >2 fold change in gene expression in a Δ*rpoS* mutant or in a Δ*bosR* mutant with an inducible *rpoS* construct relative to the parent in Caimano et al 2019 and Grassmann et al 2023 are indicated with a “Y”. Those that do not meet that criteria are indicated with an “N” and genes not included in those analyses are marked as “unknown” (21, 28). Tabs show the comparisons between the ΔBasA mutant relative to the parent, the ΔBasA mutant relative to the BasA chromosomal complement, and the BasA chromosomal complement relative to the parent.

**Supplemental Table 3. RNA sequencing mapping statistics.** All samples submitted for RNA sequencing are listed with total paired reads value depicted. The number of paired-end read pairs, total number of reads, total base pairs exceeding a quality score of 30, and the percentage of base pairs exceeding a quality score of 30 are listed. 95% of all base pairs exceeded a quality score of 30 (Q30). The total number of unique reads for each sample that map to the *B. burgdorferi* B31 reference genome are listed, broken down by borrelial plasmid associated with the reads.

**Supplemental Table 4. Proteins with an absolute log_2_ fold change >1 in TMT analysis comparisons.** Proteins depicted map to *Borrelia burgdorferi* B31 proteome. Nomenclature is based on new and old locus tag designations derived from *B. burgdorferi* B31 RefSeq and UniProt. Proteins listed met the criteria for statistical significance. Proteins associated with BosR-RpoS regulation defined as a >2 fold change in their gene expression in a Δ*rpoS* mutant or in a Δ*bosR* mutant with an inducible *rpoS* construct relative to the parent in Caimano et al 2019 and Grassmann et al 2023 are indicated with a “Y”. Those that do not meet that criteria are indicated with an “N” and genes not included in those analyses are marked as “unknown” (21, 28). Tabs list the comparisons between the ΔBasA mutant relative to the parent, the ΔBasA mutant relative to the BasA chromosomal complement, and the BasA chromosomal complement relative to the parent.
